# Cardiac resident macrophages: the emerging role in arrhythmogenesis

**DOI:** 10.3389/fimmu.2026.1753815

**Published:** 2026-02-02

**Authors:** Jiaqian Zhao, Jun Liu, Ying Zou, Jianhong Li, Ming Lei, Xiaoqiu Tan, Tangting Chen

**Affiliations:** 1 Key Laboratory of Medical Electrophysiology of the Ministry of Education Medical Electrophysiological Key Laboratory of Sichuan Province, Institute Southwest Medical University, of Cardiovascular Research, Sichuan, China; 2 Department of Physiology, School of Basic Medical Sciences, Southwest Medical University, Luzhou, Sichuan, China; 3 Department of Cardiology, The Affiliated Hospital of Southwest Medical University, Luzhou, Sichuan, China; 4 Department of Pharmacology, University of Oxford, Mansfield Road, Oxford, United Kingdom

**Keywords:** arrhythmia, cardiac resident macrophages, Connexin 43, electrical remodeling, ion channels, mechanosensitive channels, structural remodeling

## Abstract

Arrhythmia is a prevalent complication associated with various cardiovascular diseases. The onset of cardiac disease or injury can impair the normal function of cardiomyocytes, thereby precipitating arrhythmic events. Moreover, non-cardiomyocytes, including immune cells, may also play a contributory role in arrhythmogenesis. For instance, processes such as the infiltration of inflammatory cells that secrete pro-inflammatory mediators, fibroblast-to-myofibroblast transformation, and endothelial-to-mesenchymal transition have all been implicated in this process. Recent investigations have identified a distinct subset of resident macrophages within cardiac tissue that exhibit functional properties differing from those of bone marrow-derived macrophages. Cardiac tissue-resident macrophages (CRMs) are distinguished from bone marrow-derived macrophages by their developmental origin, transcriptomic profile, and functional traits. Beyond their canonical immune functions shared with bone marrow-derived macrophages, CRMs uniquely contribute to cardiac homeostasis by exerting direct electrophysiological modulation via ion channels and gap junctions. This constitutes a distinct mechanism underlying their role in arrhythmogenesis. Advanced methodologies, such as patch-clamp electrophysiology, high-throughput sequencing, and proteomic analyses in mammalian models, have revealed the complex electrophysiological interactions between CRMs and cardiomyocytes. While both CRMs and bone marrow-derived macrophages play roles in arrhythmia initiation and progression, existing reviews have primarily focused on bone marrow-derived macrophages. This review seeks to clarify the electrophysiological properties of CRMs and to delineate the specific mechanisms through which these cells contribute to arrhythmogenesis, thereby providing novel perspectives for the development of anti-arrhythmic therapeutic strategies.

## 1 Introduction

Arrhythmias, such as ventricular tachycardia (VT) and atrial fibrillation (AF), are associated with most heart diseases and cases of sudden cardiac death. A global study indicates that although the mortality rate from heart disease has declined over the past 50 years (1), the mortality rate related to arrhythmias has significantly increased. For example, the age-adjusted mortality rate for AF in the United States surged fourfold between 1979 and 2021 (2, 3). Projections suggest a stabilization by 2040, yet the continuous rise in AF-related deaths remains concerning, especially among younger adults. This concerning trend underscores the persistent clinical challenge of arrhythmia management, particularly in asymptomatic or high-risk individuals.

The pathogenesis of arrhythmias is complex and involves intricate mechanisms such as electrical and structural remodeling of the myocardium (4). Besides cardiomyocytes, non-cardiomyocytes, including immune cells, may contribute to arrhythmia through processes like inflammatory cell infiltration, pro-inflammatory mediator secretion, fibroblast-to-myofibroblast transformation, and endothelial-to-mesenchymal cell transition. Emerging evidence has reshaped our understanding of the cardiac cellular landscape, revealing a pivotal role for the immune system in electrophysiological homeostasis. Notably, macrophages exhibit significant heterogeneity stemming from the distinct differentiation and functions of resident tissue macrophages originating from embryonic sources and macrophages derived from bone marrow cells. Cardiac tissue-resident macrophages (CRMs) are now recognized not only for their canonical immunological functions, such as immune surveillance and phagocytosis, but also for their direct electrophysiological interactions with cardiomyocytes. CRMs express a diverse repertoire of ion channels (Kv1.3, Kv1.5, Kir2.1, Piezo1, etc.), form functional gap junctions via Connexin 43 (Cx43) (5), and modulate inflammatory and fibrotic pathways, thereby influencing cardiac conduction and excitability. Importantly, this direct electrophysiological regulatory capacity distinguishes CRMs from circulating monocytes, positioning them as unique cellular modulators of cardiac rhythm.

This review synthesizes current knowledge on the mechanisms by which CRMs contribute to arrhythmogenesis. We will explore CRMs roles in electrical conduction through ion channels and gap junctions, their regulation of inflammatory and fibrotic remodeling (6), and their potential as a novel therapeutic target for preventing and treating arrhythmias (7).

## 2 The spatial and temporal distribution differences of cardiac tissue-resident macrophages

Cardiac macrophages are classified into three primary subtypes based on their activation states: M0 differentiated macrophages, M1 pro-inflammatory macrophages, and M2 anti-inflammatory macrophages. Additionally, cardiac macrophages can be classified into two populations based on their origin: tissue-resident macrophages, which are established in cardiac tissue during embryogenesis, and bone marrow-derived macrophages, which are mobilized from hematopoietic stem cells in the bone marrow following injury. Macrophage origin is a critical determinant of cell behavior. In recent years, tissue-resident macrophages have become a research hotspot in the field of cardiovascular diseases (**Table 1**).

**Table 1:** Functional and histological classification of cardiac macrophages.

Cardiac macrophages				
Subtype	Functional Phenotype Classification		Tissue of Origin Classification	
	M0		CRM	MDM
	M1	M2		
Origin	Hematopoietic stem cell from bone marrow		Yolk sacs and fetal live, partially from endocardium	Hematopoietic stem cell from bone marrow
Function	Pro-inflammatory macrophages	Anti-inflammatory macrophages	Vascular development/heart regeneration; development and remodeling of extracellular matrix (ECM) and heart valves	Unclear, possibly injury/infection response
Expansion	Continuous replenishment with classical monocytes		Local proliferation	Continuous replenishment with classical monocytes

HDM, monocyte-derived macrophage; CRM, cardiac resident macrophages.

Cardiac tissue-resident macrophages (CRMs) originate from embryonic progenitors in the yolk sac and fetal liver during early heart development. CRMs subsequently colonize cardiac tissues and are maintained primarily through local proliferation rather than through continuous recruitment from myeloid monocytes (8–10).CRMs exhibit a unique anti-inflammatory/reparative metabolic phenotype and contribute directly to myocardial vascular regeneration through the secretion of angiogenic factors such as VEGF (10–13). The heterogeneity of CRMs is influenced by various factors, including different developmental stages of the heart, organ-specific characteristics, and anatomical variances (14). CRMs arise from hematopoietic precursors developing *in utero*, and persist through self-renewal mechanisms. The genetic fate map reveals that the heart harbors a significant population of macrophages originating from yolk sac and fetal monocyte precursors, distinguishing it as one of the few adult organs with this feature (11, 15, 16). During cardiac development, septation partitions the heart into four chambers, each exposed to unique mechanical forces (17–19). CRMs core function is to drive the formation of the four cardiac chambers by remodeling the endocardial cushions. Consequently, structural alterations potentially resulting in the formation of four independent chambers with specific phenotypes and functions of CRMs subsets (20). The application of unbiased single-cell RNA sequencing (scRNA-seq) has been instrumental in identifying and characterizing the diversity of these cellular subsets (18, 19). Rong Chen et al. discovered Embryonic and neonatal-derived- CX3CR1^+^CCR2^-^Ly6C^-^MHCII^-^ macrophage directly promoted cardiomyocyte proliferation via Jagged1-Notch1 axis and significantly ameliorated cardiac injury following myocardial infarction (20). Nathan et al. found significant indoor specificity in human CRMs, which among a total of 320 differentially expressed macrophage-related genes between the atrium and ventricle (21).

Furthermore, recent findings have revealed that the endocardium serves as an additional source of CRMs, giving rise to endocardium-derived macrophages. These cells emerge at E9.5(on the 9th embryonic day), exhibit robust phagocytic activity, and undergo *in situ* proliferation. They play a crucial role in extracellular matrix (ECM) development and cardiac valve remodeling (22). Under homeostatic conditions, the renewal and maintenance of CRMs mainly occur through local self-renewal. Conversely, during cardiac stress, CCR2^+^Ly6c^hi^ monocytes can replenish all CRMs subpopulations (23). Discrepancies in the surface markers used to characterize distinct CRMs subsets have been noted among different research groups. These variations are likely attributable to differences in experimental models, such as mouse strains and ages, as well as sampling locations within the heart. To address this inconsistency, we have consolidated the CRMs subsets identified in recent studies, encompassing those found in the human heart as well (**Table 2**).

**Table 2:** A summary of subcategorization schemes for cardiac resident macrophages across literature and investigators.

Species	Universal markers	Subtype/Maker	Distribution&Function		Reference
Mouse	CD45^+^, CD11b+, F4/80+ or CD64+	CX3CR1^+^CCR2^-^Ly6C^-^MHCII^-^(MP1)	MP1 promoted cardiomyocyte proliferation, Participate in the process of inflammatory response,MP2/3cluster Show a relatively stable state throughout the entire lifecycle. MP4 clusters are highly expressed in the hearts of adult mice		(11, 20, 82, 98, 110, 130, 135)
		CX3CR1^lo^CCR2^lo^Ly6C^-^MHCII^-^ (MP2)			
		CX3CR1^-^CCR2^+^Ly6C^+^MHCII^-^(MP3)			
		MHCII^+^ (MP4)			
		TLF^+^CRMs, MHCII^+^CRMs, CCR2^+^CRMs	MP1/2, MP3, and MP4 may correspond to the TLF^+^, CCR2^+^, and MHCII^+^CRMs subsets, respectively.		(136)
	/	MerTK^+^Ly6C^-^MHCII^hi^CX3CR1^hi^CD206^int^CCR2^-^	Under steady-state conditions, CRMs are renewed primarily by local proliferation. Conversely, under cardiac stress, CCR2^+^Ly6Chi monocytes can replenish all the four subsets		(11, 97) (105, 106)
		MerTK^+^Ly6C^-^MHCII^lo^CX3CR1^int^CD206^hi^CD11c^lo^CCR2^-^			
		MerTK^+^Ly6C^+^MHCII^hi/lo^ CX3CR1^hi^CD206^hi/int^CD11c^hi/lo^CCR2^-^			
		MerTK^-^Ly6C^+^MHCII^-^CX3CR1^-^CD11c^lo^CD206^-^CCR2^+^			
	/	CCR2^+^MHC-II^hi^	CCR2^+^MHC-II^hi^ macrophages fill the heart shortly after birth and maintain it through recruitment and proliferation of monocytes	CCR2^+^ macrophages are usually found in the trabecular projections of the heart, and their role in cardiac development has not been fully delineated	(90, 99, 104, 107, 108, 110, 115, 137–140)
		CCR2^-^MHC-II^hi^TIMD4^-^LYVE1^-^	/	CCR2^-^CRM occupy the myocardial wall and play a principal role in normal coronary development and maturation.	
		CCR2^-^MHC-II^hi^ TIMD4^+^LYVE1^+^	Likely derived from CCR2^-^MHC-II^lo^ macrophages during postnatal development and partially maintained by CCR2^+^MHC-II^hi^ macrophages		
		CCR2^-^MHC-II^lo^ TIMD4^+^LYVE1^-^	The most dominant subset		
		CCR2^-^MHC-II^lo^ TIMD4^+^LYVE1^+^	/		
Antenatal mice	CD45^+^, CD11b^+^, F4/80^+^ or CD64^+^, CCR2^-^MHCII^lo^	CD206^+^CRMs	Located in the subendothelial co-fusion area	The role of CRM in physiological states is not fully determined, and they may help maintain valve homeostasis, extracellular matrix remodeling, and injury repair	(141)
		MHCII^+^ CRMs	At the distal end of the aortic valve and mitral leaflet		(142)
		Gata6^+^ macrophages	The mouse pericardial cavity, the transcription curve of Gata6^+^pericardial CRM is similar to that of macrophages in the peritoneum and thoracic cavity		(143, 144)
Human	CD14^+^, CD45^+^ and the CD64^+^	MerTK^+^CCR2^-^HLA-Dr^hi^	The CCR2^+^ CRM cells are replenished by both local proliferation and recruitment of the Ly6C^hi^ CCR2^+^monocytes; they ultimately replace the yolk sac- or foetal liver-derived CRMs with ageing.		(13, 121, 135)
		MerTK^+^CCR2^+^HLA-DR^hi^			
		MerTK^-^CCR2^+^HLA-Dr^lo^			

CCR2, C–C chemokine receptor 2; CD14, cluster of differentiation 14; CD64, cluster of differentiation 64; CD68, cluster of differentiation 68; CX3CR1, chemokine (C-X3-C motif) ligand 1; HLA-DR, human leukocyte antigen DR; LYVE1, lymphatic vessel endothelial hyaluronan receptor-1; MERTK, Mer tyrosine kinase; MHC, major histocompatibility complex; TIMD4, T cell immunoglobulin and mucin domain containing 4.

**Figure 1:**
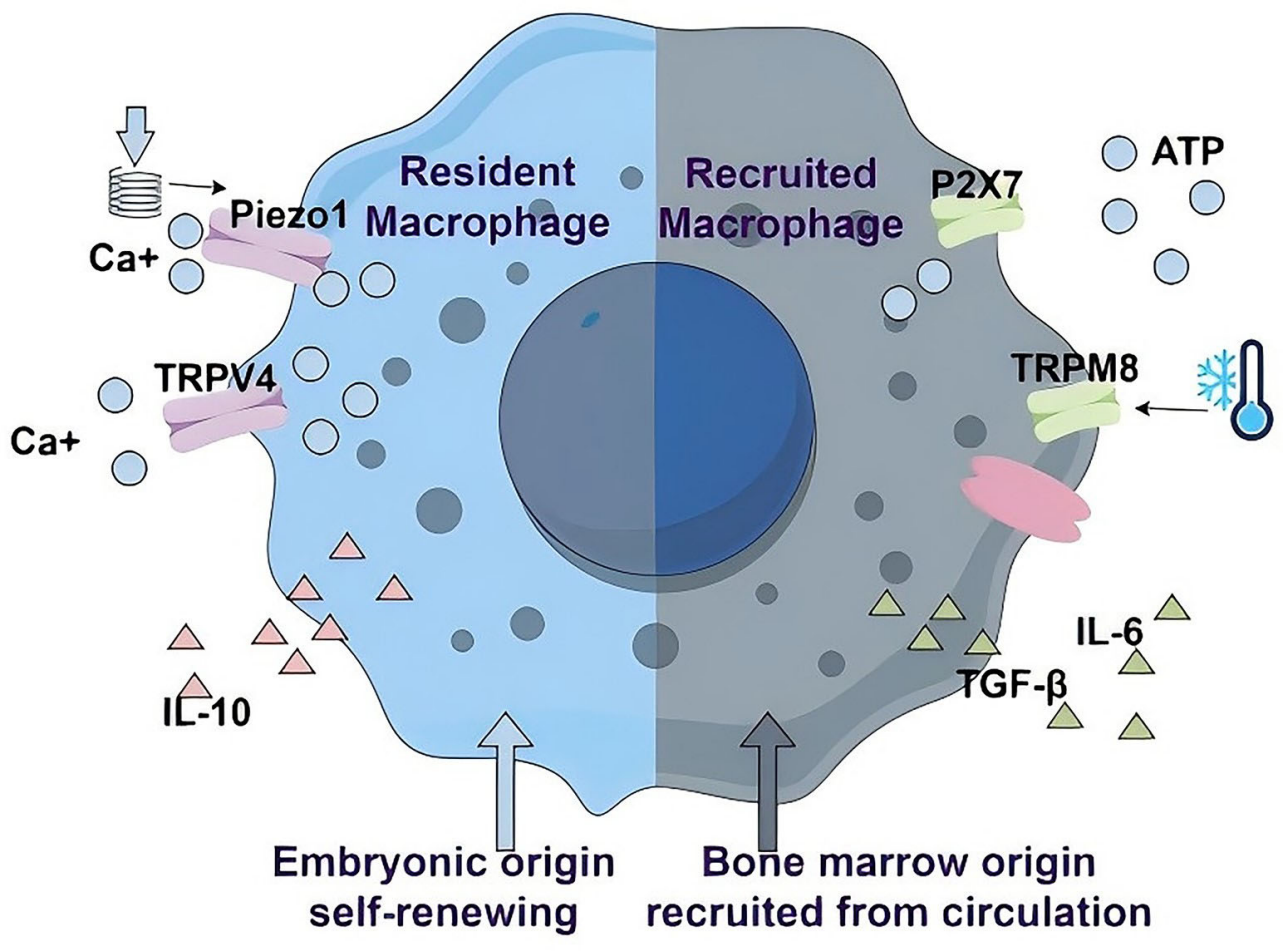
The differences between resident macrophages and recruited macrophages. Resident macrophages are of embryonic origin and self-renewing, characterized by Piezo1 and TRPV4 expression, and releasing factors like IL-10. Recruited macrophages originate from bone marrow and are recruited from circulation, expressing P2X7 and TRPM8, and releasing factors like ATP and IL-6. (TRPV4: Transient Receptor Potential Cation Channel Subfamily V Member 4. Piezo1: Piezo Type Mechanosensitive Ion Channel Component 1. IL-10: Interleukin-10. P2X7: P2X Purinoceptor 7. ATP: Adenosine Triphosphate. TGF-β: Transforming Growth Factor-Beta. TRPM8: Transient Receptor Potential Cation Channel Subfamily M Member 8).

In addition to the distinct localization of macrophages, the differential expression of ion channels contributes to the functional disparities between CRMs and bone marrow-derived macrophages (10, 24). Voltage-gated channels, such as Kv1.3, CaV1.2, and CaV1.4, are present in both cell types and influence the resting membrane potential and excitability (25), However, CRMs appear to rely more heavily on transient receptor potential (TRP) channels, including Piezo1, TRPV4, TRPC3, to detect temperature changes and mechanical stimuli, thereby specifically influencing cardiac disease states (24, 26, 27). In contrast, bone marrow-derived macrophages may depend more significantly on the P2X7 receptor and TRPM8 channel to mediate immune responses (28)(**Figure 1**). Variations among CRMs subtypes and their distinct ion channel expression patterns likely play a critical role in the pathogenesis of arrhythmias. Consequently, this review aims to provide a detailed overview of the specific mechanisms by which CRMs contribute to the occurrence of arrhythmias (26, 29, 30).

## 3 Overview of the mechanism of arrhythmia occurrence

### 3.1 Electrophysiological remodeling

Cardiac electrical remodeling encompasses alterations in action potential duration (APD), conduction velocity, ion channel function, and the coupling between cardiomyocytes. These changes are primarily associated with abnormal functionality in ion channels and structural modifications in ion channels and gap junctions (31–33). APD refers to the total time span during which a cardiomyocyte undergoes depolarization and subsequent repolarization, returning to its resting membrane potential (34). Pathological cardiac remodeling can lead to abnormal afterdepolarizations that trigger arrhythmias, such as delayed afterdepolarizations (DADs), which occur after repolarization has finished, and early afterdepolarizations (EADs), which occur during a disruption in the repolarization process. The mechanisms underlying conduction abnormalities primarily involve electrical heterogeneity as a trigger and reentry circuits as the underlying substrate (35). Disturbances in channels conduction or dynamics caused by mutations, ligand binding, or post-translational modifications can lead to minor adjustments in the balance of cell type-specific action potential currents, potentially resulting in focal ectopic activity that can serve as a trigger for arrhythmias (36). Ion channels, including sodium, potassium, and calcium channels, play crucial roles in depolarization, repolarization, and calcium influx in cardiomyocytes. The proper functioning of both the heart’s conduction system and cardiomyocyte activity relies on the coordinated activity of these ion channels. Dysfunction in any of these channels can lead to arrhythmias. Additionally, alterations in gap junctions, specialized intercellular connections that facilitate the direct passage of ions between cells, are intimately associated with arrhythmias (36, 37). These alterations may result from the phosphorylation status of gap junction proteins, and changes in key regulators may result in defects in electrical excitation or abnormalities in electrical channels, potentially leading to arrhythmias and sudden cardiac death. Beyond gap junctions, several mechanosensitive channels play a role in coordinating cardiac remodeling and survival by sensing mechanical stimuli. The recruitment and upregulation of these channels may contribute to prolonged APD and ventricular arrhythmias in cardiomyocytes (38).

### 3.2 Structural remodeling

Pathological cardiac structural remodeling encompasses compensatory hypertrophy of cardiomyocytes, necrosis and apoptosis, along with the proliferation and migration of non-cardiomyocytes, alterations in extracellular matrix components, and myocardial fibrosis. These changes ultimately lead to alterations in the size, shape, wall thickness, and function of the heart, adversely affecting the heart’s electrical conduction properties, ventricle overload, and resulting in the occurrence of arrhythmias.

The remodeling of cardiomyocytes includes hypertrophy, apoptosis, and changes in cell morphology, all of which can affect the electrophysiological properties of the heart. Cardiomyocyte hypertrophy alters the surface area and volume of the cell membrane, affecting the distribution and function of ion channels, which in turn impacts the formation and conduction of action potentials in cardiomyocytes. Apoptosis of cardiomyocytes further reduces the number of normal cardiomyocytes, exacerbating the heterogeneity of electrical conduction. Changes in gap junctions of cardiomyocytes, such as the remodeling of Cx43, are an important component of cardiac structural remodeling. Abnormal expression and distribution of Cx43 can lead to impaired electrical coupling between cardiomyocytes, increasing the risk of arrhythmias.

Myocardial interstitial remodeling involves alterations in the structure and function of myocardial interstitial cells and the extracellular matrix. On one hand, inflammatory factors stimulate the activation of fibroblasts, which then transform into myofibroblasts that secrete large amounts of collagen (39). On the other hand, an imbalance between matrix metalloproteinases (MMPs) and their inhibitors, metalloproteinases (TIMPs), ultimately leads to excessive deposition or degradation of the extracellular matrix (40, 41). This remodeling disrupts normal electrical conduction pathways between myocardial cells, increasing heterogeneity and delaying electrical signal propagation, thereby facilitating the emergence of reentrant arrhythmias (42).

## 4 CRMs participate in the occurrence of arrhythmia

The CRMs not only possess typical immune cell functions, such as immune surveillance, antigen presentation, and immune phagocytosis, but also play unique electrophysiological roles. In recent years, advanced technologies such as patch-clamp techniques, transcriptome sequencing, and proteomics have been used to study mammalian hearts, revealing electrophysiological interactions between CRMs and cardiomyocytes. The subsequent review delineates the mechanisms through which CRMs modulate arrhythmias.

### 4.1 CRMs participate in the occurrence of arrhythmia through ion channels

#### 4.1.1 Potassium channel

A mouse model was employed to characterize the passive and active electrophysiological properties of mouse CRMs. Utilizing ion channel blockers, flow cytometry, immunostaining, and RNA sequencing, the authors identified the primary voltage-gated potassium (K^+^) channels underlying the electrophysiological behavior of CRMs as Kv1.3, Kv1.5, and Kir2.1. The computational model was also established to describe the electrophysiological characteristics of mouse CRMs. These channels (Kv1.3, Kv1.5, Kir2.1) were instrumental in observing distinct ion currents. CRMs exhibited four modes of outward rectification and two modes of inward rectification of potassium currents. RNA-seq data confirmed the stable expression of genes encoding potassium channel proteins—including Kcna3, Kcna5, Kcng2, and Kcnj2 (coding Kv1.3, Kv1.5, Kv6.2, and Kir2.1 channels)- are stably expressed in CRMs (43, 44). Although RNA-seq indicated high mRNA levels for calcium channel genes, no functional evidence currently supports the presence of voltage-activated calcium channels in CRMs. Kv1.3 and/or Kv1.5 are considered the primary mediators of outward current, while Kir2.1 mediates inward-rectifying K^+^ currents in cardiac-resident macrophages. The Kcng2 gene encodes the Kv6.2 channel, which is electrophysiologically silent on its own but can form heteromers with Kv2.1 channels, thereby altering their voltage-dependent activation and deactivation kinetics (45–48). Furthermore, cytokines (IL-1β, TNF-α, TGF-β, IL-6) can modulate potassium current density, thereby altering atrial conduction speed and atrial effective refractory period (AERP), which increases susceptibility to atrial fibrillation (AF) (28, 49, 50). Recent studies have shown upregulated expression of KCNN4 in macrophages recruited to the heart after myocardial infarction. KCNN4 encodes calcium-activated potassium channel KCa3.1, which maintains the negative membrane potential required for continuous Ca^2+^ influx (51, 52). In the infarction border area, increased KCa3.1 channel activity in recruited macrophages promotes Ca²^+^ influx, further leading to APD prolongation in cardiomyocytes and enhancing susceptibility to ventricular arrhythmias (52). Therefore, it is hypothesized that monitoring changes in the velocity and intensity of current signals during arrhythmias could help explore the anomalies of specific gene-encoded channels.

#### 4.1.2 Piezo1 channel

**Figure 2:**
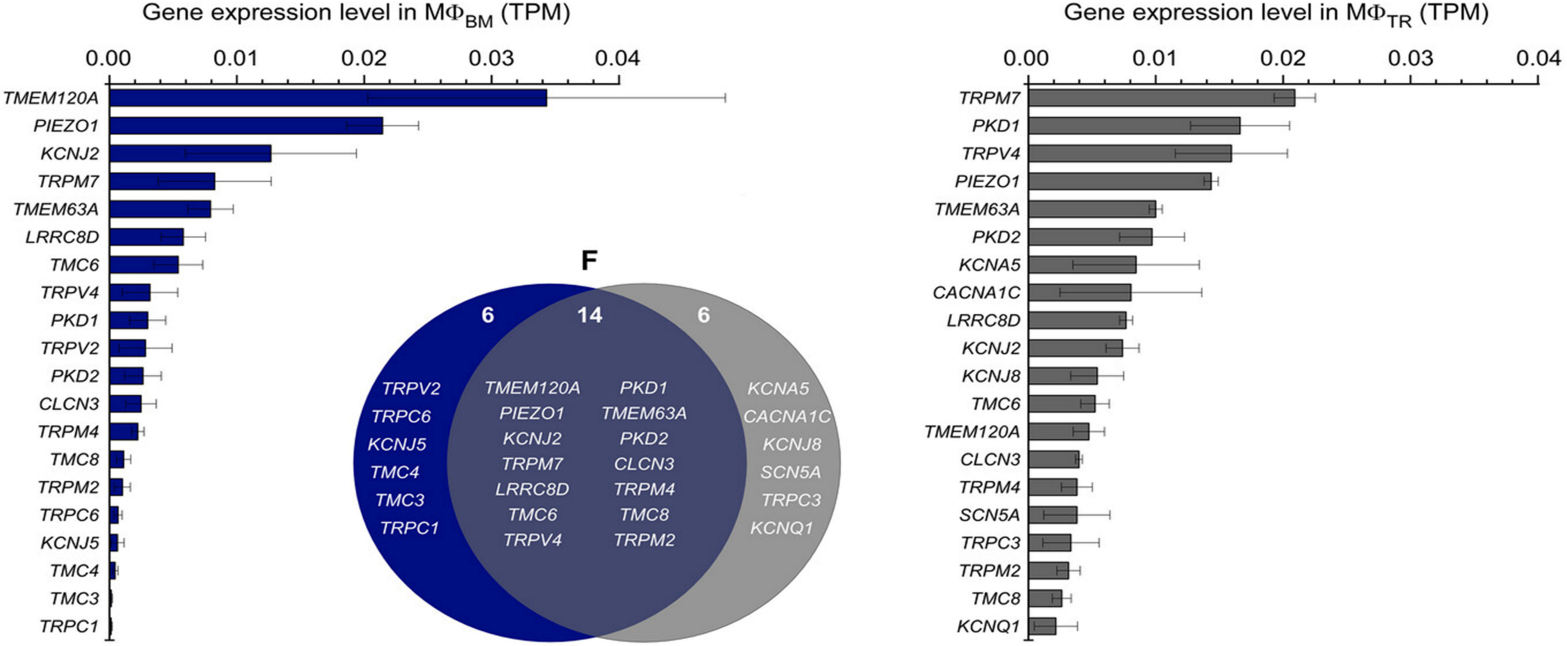
Differential expression of piezo1 in bone marrow-derived and tissue resident macrophages (Image adapted from: Piezo1 stretch-activated channel activity differs between murine bone marrow-derived and cardiac tissue-resident macrophages. J Physiol. 2024 Sep;602(18):4437-4456. doi: 10.1113/JP284805. Epub 2024 Apr 20. PMID: 38642051.).

The mechanosensitive non-selective cation channel Piezo1 is widely expressed in various cell types across species and participates in numerous biological processes (53–56). It has been demonstrated to play a regulatory role in immune cell activity (57). In macrophage-specific Piezo1 knockout mouse models, gene deletion of Piezo1 has been shown to reduce inflammation and promote wound healing (57–59). Recent evidence indicates that Piezo1 is involved in regulating the development and progression of myocardial fibrosis, atherosclerosis, and other cardiovascular diseases (60). Furthermore, studies by Solis, Bielecki et al. have demonstrated that Piezo1 functions as a signaling pathway capable of inducing inflammatory gene expression in mouse monocytes. Mice deficient in Piezo1 in bone marrow-derived cells exhibited reduced pulmonary inflammation during bacterial infection or fibrotic autoinflammation (61, 62). The combination of the above experiments’ evidence indicates that the expression and/or activity of Piezo1 can fundamentally modulate core functional responses in macrophages. To identify the molecular basis of the observed stretch-induced currents, researchers analyzed RNA expression levels in freshly isolated macrophage populations and performed comparative analyses. Both bone marrow-derived macrophage and CRMs revealed the expression profiles of genes encoding mechanosensitive channels (**Figure 2**). Among these, Piezo1 was one of the most highly expressed genes across both subsets, underscoring its central role in macrophage mechanotransduction. These findings confirm that Piezo1 serves as the dominant channel mediating mechanosensation in bone marrow-derived macrophages, primarily through Ca²^+^ signaling that regulates metabolic reprogramming and inflammatory responses (27, 63, 64).

Piezo1, as a mechanosensitive ion channel, detects changes in mechanical stress within the extracellular matrix (ECM). In cardiac tissue, it plays a key role in sensing mechanical interactions between cardiomyocytes and heart wall tension, while also regulating the influx of calcium ions (Ca^2+^) into cardiomyocytes, which are essential for the electrophysiological activities of cardiomyocytes. Abnormal Piezo1 activity may lead to cellular calcium overload, resulting in instability of the cardiomyocyte membrane potential, aberrant excitation, and conduction disturbances. These abnormalities facilitate reentrant excitation circuits—a central pathophysiological basis for various types of arrhythmias (65–67). Upon activation, Piezo1 triggers calcium-dependent signaling pathways, influences the function of CRMs, promotes the release of inflammatory factors, and modulates action potential duration (APD) and conduction velocity, leading to electrical and structural remodeling of the heart and ultimately contributing to the development of arrhythmias (67, 68).

Under clear pathological conditions, particularly following myocardial infarction, abnormal activation of Piezo1 in macrophages has been confirmed to drive malignant cardiac remodeling and arrhythmias. For example, Tao Ling’s team discovered that after myocardial infarction, myeloid-specific deletion of Piezo1 can reduce the upregulation of SLC7A11 via the calcium/ATF4 pathway, thereby decreasing the impaired phagocytic function of macrophages, which in turn slows the progression of inflammation, fibrosis, and electrical instability (69). Similarly, under chronic pressure overload, Piezo1 can act as a pathological mechanical signal sensor, driving macrophages toward a pro-inflammatory phenotype and promoting the release of IL-6, further worsening the arrhythmogenic substrate (70). Conversely, under physiological or mild pathological conditions, moderate activation of Piezo1 helps maintain tissue mechanical homeostasis, suppresses excessive inflammation, and supports repair, thereby exerting a protective effect. Notably, there are critical differences among macrophage subsets: bone marrow-derived macrophages exhibit Piezo1 channel activity, whereas no such activity has been detected in cardiac tissue-resident macrophages, suggesting that their function highly depends on the cellular source (27).

Therefore, the role of Piezo1 in the occurrence of arrhythmias exhibits significant context dependency, demonstrating a dual and paradoxical effect of both promoting and suppressing arrhythmias. In the early/stable phase of injury, Piezo1 on CRMs senses environmental mechanical forces (such as tissue deformation in the initial stage of injury), with a protective effect predominating. In the middle to late/chronic phase of injury, Piezo1 on a large number of bone marrow-derived macrophages senses pathological mechanical environments (such as abnormal stiffness in fibrotic areas after myocardial infarction), driving pathological remodeling. Future research should focus on elucidating the mechanisms by which Piezo1 contributes to specific subtypes of arrhythmias, thereby providing a theoretical foundation for developing targeted therapeutic strategies (71–73).

#### 4.1.3 Other channels

In addition to expressing voltage-gated potassium channels, CRMs also express a variety of other ion channels, including TRPV4, TRPM7, and TRPC3. Wong et al. indicate that CRMs coordinate adaptive remodeling and promote survival of the failing heart through the TRPV4-dependent mechanism of mechanosensation (10). Expression analysis of known mechanical reaction factors revealed high levels of TRPV4 mRNA in CCR2^+^ macrophages. Proportional calcium assays confirmed TRPV4 channel activity in CRMs, a finding further supported by immunofluorescence staining and flow cytometry experiments conducted in transgenic mice, which demonstrated predominant expression of TRPV4 in CRMs and neutrophils. Furthermore, *in situ* detection of TRPV4 activity revealed an increased cytoplasmic calcium concentration in CRMs. Controlled experiments further indicated that TRPV4 independently promotes the production of macrophage-derived factors in response to mechanical stretch, suggesting that mechanosensation represents one key mechanism by which CRMs are activated in the failing heart (10). Following the onset of arrhythmia, alterations in current density, variations in speed of electrical signal transmission, and changes in APD become critical electrophysiological determinants. Tracing the genes of the coding ion channels, as well as the channels or subunits in related expression, has become the key node for subsequent exploration of heart runaway diseases and arrhythmias (74).

Tom Schilling et al. initially identified TRPM7 in macrophages, indicating its involvement in the polarization of macrophages towards the M2 phenotype and the proliferation of M2 macrophages (75). Subsequently, Francisco J et al. further studied the role of TRPM7 in the heart and kidneys through the use of specific peritoneal resident macrophage TRPM7 knockout mice, demonstrating that TRPM7 can prevent inflammatory reactions and fibrosis in the cardiovascular system, and suggesting that its protective effects are partly mediated by Mg^2+^-sensitive processes (76). Nevertheless, the specific mechanisms and functions of TRPM7 in cardiac resident macrophages (CRMs) remain an important area for future exploration.

TRPC3 plays a significant role in regulating Ca²^+^ influx, which is crucial for both physiological functions and the pathogenesis of various cardiovascular diseases (63, 64). A substantial body of experimental evidence supports the involvement of TRPC channels, including TRPC3, in signal transduction mechanisms related to primary hypertension, cardiac hypertrophy, intimal hyperplasia, and endothelial dysfunction. Furthermore, studies have indicated that TRPC3 can influence the apoptosis rate of macrophages under disease conditions, thereby potentially affecting the incidence and progression of cardiovascular pathologies (77). However, the expression of TRPC3 is widespread, and macrophages are not the only cells that possess this channel. Additionally, there are numerous factors involved in the development of lesions *in vivo*, and the study of TRPC3 in CRMs has not yet been explored. In summary, while TRPC3 is implicated in broader cardiovascular disease processes, its cell-specific mechanisms within CRMs are not yet defined, and its direct link to arrhythmogenesis requires further investigation (10, 78–80).

The mechanistic insights derived from mouse models, as summarized above, outline how CRMs may influence the cardiac microenvironment and electrical homeostasis through specific ion channels such as TRPV4, TRPM7, and TRPC3. These findings receive direct and compelling support from human systems. A pivotal study in 2025 utilizing a co-culture model based on human induced pluripotent stem cell -derived cardiomyocytes (hPSCs-CM) and CRMs, demonstrating for the first time *in vitro* that human macrophages can directly modulate calcium transients, APD, and contraction. Ion channel dot plot profiling showed the expression of calcium and potassium channels in CRMs, including CACNA1F, KCNA3, KCNJ10, KCNMA1, KCNN4, TRPA1, TRPC2, TRPM2, TRPV2, and TRPV4. Crucially, the presence of CRMs significantly influenced the pro-arrhythmic phenotype of cardiomyocytes, providing direct evidence that paracrine extracellular vesicles and contact-dependent interactions between CRMs and cardiomyocytes constitute a key node in arrhythmogenesis (81).

In the investigation of sepsis-induced cardiomyopathy, the integration of scRNA sequencing and genetic fate-mapping technologies has enabled the identification of a distinct subpopulation of cardiac-resident macrophages—denoted as CD163^+^RETNLA^+^ (Mac1)—which exhibits the capacity for self-renewal during sepsis (82). This subgroup has the characteristics of an endogenous transcriptome and high expression characteristics of TREM2, and the Trem2 ablation damages the self-renewal ability of Mac1 cells, and the highly expressive Mac1 cells of TREM2 improve heart function. Based on the close influence of its cells on heart function, this may become another new direction for exploring arrhythmias (82, 83).

Despite these facts, much of our understanding of the human CRMs’ ion channels is derived from animal models, which often fail to accurately replicate the physiology of the human heart. This discrepancy results in inadequate translation to clinical applications. Consequently, there is a need for further research on the ion channel characteristics of CRMs and their effects on the electrical activity of cardiomyocytes, utilizing human pluripotent stem cells and/or human cardiac organoid systems.

### 4.2 CRMs participate in the occurrence of arrhythmia through Cx43

CRMs are particularly enriched at the distal region of the atrioventricular node (AVN). These cells express Cx43 and form functional gap junctions with cardiomyocytes, thereby directly modulating electrical conduction within the heart (15). In adults, CRMs contribute to the regulation of atrioventricular function via Cx43-containing gap junctions, enabling electrotonic coupling between macrophages and spontaneously beating cardiomyocytes (84). CRMs are coupled with spontaneously beating cardiomyocytes, have negative resting membrane potentials, and depolarize synchronously with cardiomyocytes. Optogenetic stimulation of macrophages expressing channelrhodopsin-2 (ChR2) enhances atrioventricular conduction. Conversely, conditional knockout of Cx43 (Cx3cr1 Cx43^−/−^) in resident macrophages or using CRMs-induced ablation mice (Cd11b DTR) leads to a delay in AV conduction and can even cause atrioventricular conduction block (84). These findings underscore the critical involvement of CRMs in the electrophysiological regulation of the heart and highlight their potential contribution to arrhythmogenesis.

Normal gap connection activates cardiomyocytes’ action potential through Cx43, but changes in gap connection coupling occur with various forms of heart diseases. These coupling changes will lead to electrical excitation defects, resulting in malignant arrhythmia and sudden cardiogenic death (85, 86). Sugita conducted in-depth research and a comparison of these connecting proteins. Gap-connected intercellular communication (GJIC) is regulated by the post-translational modification of Cx43, which directly controls channel activity or regulates protein-protein interaction to localize with Cx43 (85). Phosphorylation is the most adequate modification. Many studies have shown that it plays an important role in regulating channels-gate control, transportation, assembly/disassembly, and degradation of gap junction channels (87). Moreover, Cx43 dephosphorylation is a characteristic of ischemia, arrhythmia, and heart failure/aging. Sugita found that AREG has been identified as a key regulator of Cx43 phosphorylation and localization. In contrast, AREG knockout mice (Areg^-/-^) exhibit isoproterenol-dependent VT (88) and showed an increase in Cx43 phosphorylation and obvious Cx43 partial lateralization, which led to an increase in arrhythmia and post-pulmonary artery bundle mortality. Treatment with recombinant AREG restored Cx43 phosphorylation and localization, preventing sudden cardiac death (SCD) (88).

In addition, CRMs also exhibit surface expression of Cx43, which is essential for both homotypic and heterotypic electrotonic coupling with cardiomyocytes. Cora et al. utilized co-cultures of murine HL-1 cardiomyocytes and RAW 264.7 macrophages to maintain macrophages in M2c anti-inflammatory phenotypes via cytokine treatment with TGF-β1 and IL-10 to mimic CRMs. It was found that TGF-β1 and IL-10 upregulated the Cx43 semi-channel, thus enhancing macrophage- cardiomyocytes coupling, increasing the cell resting membrane potential, and leading to more excited cardiomyocytes (43). Conversely, in the context of myocardial infarction, pro-inflammatory mediators derived from macrophages have been shown to promote Cx43 degradation. Matrix metalloproteinase-7 (MMP-7) processes Cx43 at its C-terminal site, leading to reduced ventricular Cx43 levels and impaired conduction velocity, thereby increasing susceptibility to arrhythmias and sudden cardiac death. Similarly, interleukin-1β (IL-1β) contributes to excitation-contraction uncoupling and arrhythmogenesis through Cx43 degradation post-infarction (89–91). The potassium channel KCa3.1 in the macrophages recruited in the infarction boundary area is up-regulated to promote the inflow of Ca^2+^ to macrophages, and the elevated intracellular Ca^2+^ then flows from the recruited macrophages to neighboring myocardial cells through Cx43, which leads to the prolongation of myocardial cell APD. Xin et al. propose that macrophage migration inhibitory factor is implicated in the pathogenesis of patients with chronic atrial fibrillation, likely through the down-regulation of protein and gene expression of Cx43 via the activation of ERK1/2 kinase. In the context of myocardial infarction, activated pro-inflammatory macrophages secrete factors like IL-1β and MMP7, which directly contribute to Cx43 degradation and gap junction remodeling in cardiomyocytes, thereby increasing arrhythmic susceptibility (92). This process is regulated by macrophage-intrinsic signaling, such as miR-155, linking immune activation to conduction disturbance. Targeting this macrophage-cardiomyocyte crosstalk has emerged as a therapeutic strategy (93, 94). For instance, a novel conductive polymer has been shown to suppress MAPK signaling, restore Cx43 expression, and alleviate AF in models, highlighting a material-based approach to correct this deficit (95). Furthermore, studies using hPSCs-CMs are pivotal for validating these targets in a human context (96). Therefore, for the repair and maintenance post-arrhythmia, ensuring the integrity of Cx43 phosphorylation, the correct construction and conduction of channel connections, and the precise regulation of cytokines will be the focus of subsequent research on Cx43 in arrhythmia.

All these findings show that CRMs promote cardiac electrical conduction through the electrical coupling of Cx43 with myocardial cells. Cx43 plays a role in the connection between cardiomyocytes, which helps electrical signals spread quickly and accurately between cardiomyocytes. Mutation or abnormal conduction or functional blockage of Cx43 may affect signal conduction between cardiomyocytes. Abnormal coupling, dephosphorylation, and abnormal partial lateralization may cause delay or interruption of electrical signal conduction, interfere with the normal rhythm of the heart, affect the function and electrical activity of the heart, and even influence the infarction area.

### 4.3 CRMs combat arrhythmogenic remodeling

During the inflammatory response, chemokines (CC and CXC), recruited monocytes, and neutrophils are significantly associated with myocardial fibrosis. The contractile activity of fibrotic cardiac tissue is inhibited, allowing inflammation to propagate and cause rhythm disorders (6).CRMs possess the ability to modulate inflammatory responses, which confers a protective effect against arrhythmias.

Conversely, immune responses activate cardiac fibroblasts. Uncontrolled tissue damage and sustained pro-inflammatory signaling lead to unregulated extracellular matrix production, promoting cardiac fibrosis and hypertrophy, and causing defects in the cardiac conduction system (97). Myocardial fibrosis and dying or dead cells may also slow down conduction, thereby promoting arrhythmia (98). Elucidation of the functional mechanisms of CRMs in pathological cardiac inflammation and fibrogenesis can be achieved, which would unveil novel therapeutic targets for arrhythmogenic disorders. Consequently, the following analysis will delineate these mechanisms through the CRMs: their anti-inflammatory actions and anti-fibrotic interventions in myocardial remodeling.

#### 4.3.1 CRMs mediating inflammation

The research results have clearly confirmed that CRMs play a key molecular regulatory role in regulating inflammation resolution and promoting the recovery of damaged cardiac function (99). CRMs mainly alleviate cardiac injury by secreting cytoprotective factors (such as cytokines, chemokines, and growth factors), eliminating damaged cells or mitochondrial remnants, and regulating cardiac conduction, angiogenesis, lymphangiogenesis, and fibrosis, etc. (100).

When inflammation occurs, inflammatory cells tend to accumulate in large numbers. These cells downregulate anti-inflammatory cytokines such as interleukin-10 (IL-10) and transforming growth factor-β (TGF-β), while upregulating pro-inflammatory mediators such as tumor necrosis factor-a (TNF-α), IL-1β, IL-6, and IFN-γ (49). Overexpression of TNF-α prolongs APD, significantly reduces the repolarization potassium current and delayed calcium reuptake, while the L-type Ca^2+^ current (I_CaL_) remains unchanged, accompanied by a decrease in the expression of the SR Ca^2+^ ATPase (SERCA) gene. TNF-α and IL-1β have similar effects on intracellular calcium handling. When combined, they act synergistically to further decrease systolic [Ca^2+^], decrease [Ca^2+^] SR, and increase spontaneous SR Ca^2+^ release events, leading to arrhythmogenic Ca^2+^ waves. Some reports indicate that IL-1β reduces the expression of the ryanodine receptor (RYR), SERCA, and phospholamban (PLB). IL-1β can also cause intercellular uncoupling, internalization, and a decrease in Cx43 expression. IL-6 signaling through gp130 (glycoprotein 130 cytokine receptor) leads to an increase in I_CaL_, an increase in systolic [Ca^2+^], and an extension of APD. IL-6 has also been shown to reduce SERCA gene expression and PLB phosphorylation, resulting in a slower rate of calcium reuptake into the SR. These proteases, MMPs, may also degrade gap junction proteins required for cell electrical coupling (6).

Therefore, studying the participation and protective mechanisms of CRMs in pathological inflammatory responses is crucial for understanding their contribution to abnormal cardiac functions, such as arrhythmias.

For instance, during the occurrence of AF, IL-1β-induced AF sensitization depends on the presence of IL-1Rs on CRMs, rather than on cardiomyocytes, as well as on the caspase-1/IL-1β axis. This suggests that CRMs transduce signals into soluble, membrane-bound, or electrical signals and convey them to cardiomyocytes. Studies also demonstrated that CRMs are crucial for mediating IL-1β-induced shortening of APD and triggering activity (101).

During myocardial infarction, CRMs promote cardiomyocyte proliferation and angiogenesis after myocardial injury through local proliferation and the production of high levels of growth factors in neonatal mice (102). Studies have shown that CCR2-CRMs exhibit high expression of various growth factors, including IGF1, PDGF-C, EGFL7, GDF15, NRP1, SLIT3, ECM1, SDC3, SCN9A and FGF13, etc. In adult mice, CX3CR1^+^ CRMs produce IL-10, inhibit cardiac collagen deposition by reducing hyaluronidase levels, and improve left ventricular function and promote cardiac wound healing. Recent studies have shown that damaged or dysfunctional mitochondria can be cleared through cardiac exosomes in cardiomyocytes, which are mainly captured and cleared by the MerTK receptor on CRMs (11). Mitochondrial proteins and DNA, as damage-associated molecular patterns (DAMPs), can trigger intracellular and extracellular inflammatory responses.

Some studies suggest that CCR2^-^CRMs exhibit anti-inflammatory properties by inhibiting harmful type I interferons (IFN) through the Nrf2 pathway, which is dependent on the Tet2 and Irf3 transcription factors (103). Blocking the colony-stimulating factor 1 receptor (Csf1R) can enhance the survival of CRMs and the proliferation of bone marrow cells (103, 104). Blocking the MCP-1 (monocyte chemoattractant protein) and CCR2 signaling pathways to reduce monocyte recruitment can alleviate the inflammatory response and confer cardiac protection in mouse models of myocardial infarction (105, 106). On the other hand, CCR2^+^CRMs release various cytokines and chemokines, especially MCP-1, acting as a pro-inflammatory cytokine, which can enhance the expression of inflammatory cytokines in monocytes and macrophages. Clearing CCR2^+^CRMs before cardiac injury can not only reduce monocyte recruitment but also decrease the tendency for IFN accumulation and inhibit inflammation. The depletion of CCR2^-^CRMs in tissues increases the number of Arg1 and CXCL1 clusters, driving the differentiation of recruited monocytes, which is conducive to inflammation (107). Daile et al. found that legumain (Lgmn), encoded by a gene specifically expressed in CRMs and mainly in the TIMD4^+^CCR2^−^cluster (TIMD4 is one of the persistent lineage markers of resident cardiac macrophage subpopulations), is a lysosomal enzyme essential for cell proliferation. The absence of Lgmn leads to defects in intracellular calcium mobilization in CRMs, resulting in decreased cytoplasmic calcium levels, downregulation of anti-inflammatory mediators IL-10 and TGF-β, and upregulation of pro-inflammatory mediators IL-1β, TNF-α, IL-6, and IFN-γ. The ability of CRMs to clear and degrade apoptotic cardiomyocytes is inhibited due to the lack of cytoplasmic calcium. Therefore, maintaining intracellular calcium levels and phagocytic ability in CRMs is one of the important means to protect cardiac function recovery after myocardial infarction and reduce the incidence of arrhythmias (99).

Cristina et al. found that in lipid metabolism defect-induced cardiac inflammation, the relative abundance of monocytes increases, while the relative abundance of TIMD4^+^CRMs decreases. TIMD4^+^CRMs enrich nuclear receptor signaling pathways with anti-inflammatory properties. By reshaping the phenotype of macrophages, restoring the number and homeostatic function of TIMD4^+^CRMs, and inhibiting cardiac inflammation (108), cardiac dysfunction can be alleviated, thereby preventing arrhythmias.

CRMs can recruit infiltrating macrophages under stress. CRMs and infiltrating macrophages have distinct origins and are located in different geographical positions; thus, they have different functions. Liao found that pressure overload one week after TAC would induce CCR2^−^CRMs proliferation, which was regulated by Kruppel-like factor 4. While in the late stage of pressure overload (14 days later), Ly6C^hi^ monocytes were induced to infiltrate, and the infiltrating monocytes/macrophages promoted decompensation. They believed that the loss of CRMs after TAC might lead to an increase in susceptibility to arrhythmia (109).

Diastolic dysfunction and local chronic inflammation are increased in aged mice. Xia found that increased CRM accumulation was observed in the hearts of aged mice. During aging, CCR2^+^ CRMs gradually replace CCR2^-^CRMs. The function of aged CCR2^+^CRMs shifts to a pro-inflammatory state, up-regulating the pro-inflammatory factors IL-1β and TNF-α to act on cardiomyocytes, and enhancing pathways such as cytoplasmic DNA sensing pathways, TNF signaling pathways, and complement cascades. This may be one of the factors contributing to the increased susceptibility to arrhythmias in the elderly (110).

#### 4.3.2 CRMs influence the fibrotic process

Fibrosis-induced arrhythmias have traditionally been attributed to ECM deposition in the interstitial space. The dense fibrosis of scars, predominantly acellular and electrically non-excitable, may serve as an insulating region where reentrant arrhythmias can anchor and transform into sustained ventricular arrhythmias. Interstitial fibrosis can separate myocytes from each other by creating small, electrically insulating regions between them. This electrical decoupling reduces source-sink mismatch and has been shown to promote the escape of ectopic triggers, facilitate slow conduction and unidirectional conduction block, thereby favoring reentrant tachycardia. Due to the frequent disruption of side-to-side connections between myocardial cells by interstitial fibrosis, anisotropy of conduction may also increase. As the electrical tension load on cardiomyocytes increases (i.e., current sink increases), myocyte-myocyte coupling decreases, and the difference in resting membrane potential between cardiomyocytes and myofibroblasts (Mfbs) (respectively -80 mV and -50 mV to -20 mV), the coupling of Mfbs with cardiomyocytes significantly reduces conduction velocity (CV). With an increase in Mfb density, the resting membrane potential of cardiomyocytes becomes more depolarized, inactivating voltage-gated Na^+^ channels, thereby slowing conduction and causing post-repolarization refractoriness. Furthermore, as diastolic depolarization can lower the threshold for ectopic activity, this state can also lead to arrhythmias (6).

The atrium is exquisitely sensitive to immune-interstitial crosstalk. This sensitivity underlies its susceptibility to atrial fibrillation (AF), a pathology driven by recruited CCR2^+^ monocyte/macrophage lineages—particularly the SPP1^+^ subset. These cells promote diffuse interstitial fibrosis by secreting key mediators (e.g., TGF-β1, IL-1β, TNF-α) that directly activate atrial-specific fibroblasts, thereby creating a substrate for AF initiation and persistence. CRMs internalize the mitochondrial DNA (mtDNA) and reactive oxygen species (ROS) released from injured myocardial tissue, inhibiting the activation of NLRP3. Indeed, cardiomyocyte-specific NLRP3 gain-of-function (Nlrp3^A350V/+^) mice are more susceptible to pacing-induced AF (111). CRMs can reduce the likelihood of arrhythmia in these mice.

In contrast, the ventricle’s primary function is maintaining structural integrity. Following ventricular injury, embryonically derived CRMs coordinate repair through anti-fibrotic and pro-angiogenic programs. However, dysregulated or excessive repair leads to the formation of dense scar tissue with electrophysiologically heterogeneous border zones. These areas cause conduction slowing and block, forming the anatomical substrate for re-entrant ventricular arrhythmias (29, 112). After cardiac injury, CRMs highly express MerTK and Axl receptors, actively eliminating apoptotic cardiomyocytes, blocking the necrosis-inflammation-fibrosis cascade reaction (11, 113), and preventing the release of pro-fibrotic factors such as TGF-β and IL-1α from apoptotic cells. TGF-β stimulates fibroblasts to differentiate into Mfbs, inhibiting this via macrophage activity enhances the expression of tissue metalloproteinase the expression of tissue metalloproteinase inhibitors (TIMPs) and other protease inhibitors, which may reduce fibrosis and lower the possibility of arrhythmia (114). CRMs secrete the anti-fibrotic factor IL-10, block the TGF-β/Smad pathway, inhibits the transformation of fibroblasts into Mfbs, and prevent the structural basis of arrhythmia (11, 109). Under specific conditions, CRMs secrete VEGF and IGF-1 (insulin-like growth factor 1) (115) to promote angiogenesis, improve ischemia and hypoxia, and reduce the production of core fibrotic factors. At the same time, CRMs secrete IL-10 and TGF-β to inhibit neutrophil infiltration and IL-17 (a pro-fibrotic factor), and suppress inflammation-driven fibrosis.

Xu discovered that the functional characterization obtained from the co-culture of Bhlhe41^+^CRMs with cardiomyocytes and fibroblasts, along with the depletion of Bhlhe41^+^ CRMs, highlights the significant role of Bhlhe41^+^CRMs in inhibiting the activation of myofibroblasts. This research emphasizes the crucial significance of the Bhlhe41^+^CRMs phenotype and its plasticity in reducing excessive fibrosis and limiting the advancement of the infarct region (116). Similarly, Gata6^+^CX3CR1^+^CRMs in the pericardial fluid of mice infiltrated into the epicardium and lost Gata6 expression but still showed anti-fibrotic function. However, the specific role of CRMs that initially settle in the heart is still unclear, and further research is needed. At the same time, the researchers also found that CRMs expressing LYVE1 (TLF^+^CRMs) can indirectly inhibit β-adrenergic receptor-triggered cardiac fibrotic damage (117).

In hPSCs-derived primitive yolk sac-like macrophages, it was found that CRMs significantly upregulate the expression of HGF (hepatocyte growth factor), which activates the downstream c-Met receptor signaling pathway, inhibits fibroblast activation, reduces collagen synthesis, and reduces susceptibility to arrhythmia (118).

## 5 Conclusion

**Figure 3:**
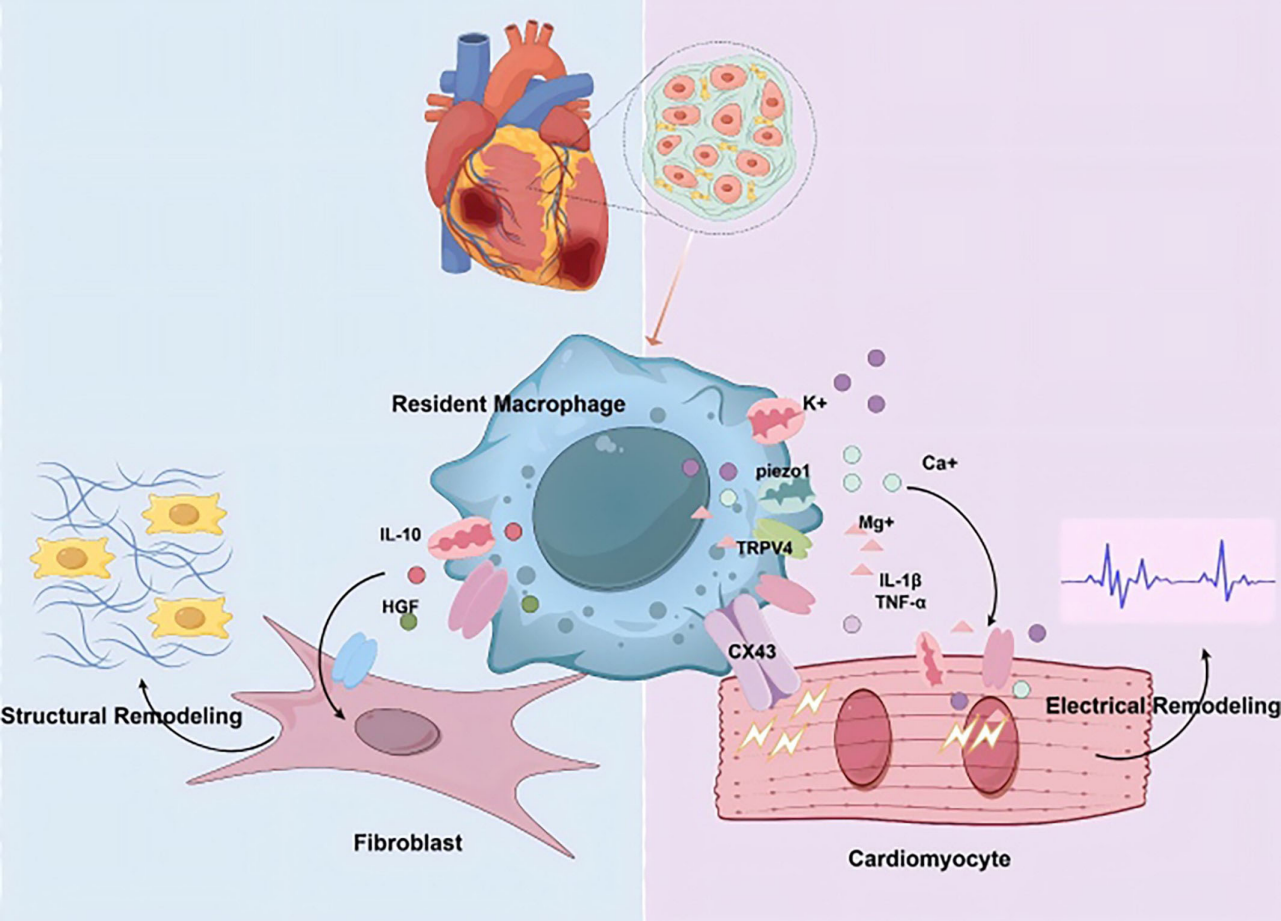
The dual mechanisms of cardiac resident macrophage in mediating arrhythmias. Cardiac resident macrophages drive structural remodeling through two primary pathways: by modulating inflammatory responses or by promoting fibrotic processes to drive structural remodeling, while also interacting with cardiomyocytes via ion channels, other channels and gap junctions to drive electrical remodeling. (Cx43:Connexin 43.HGF: Hepatocyte Growth Factor.IL-1β:Interleukin-1 Beta. TNF-α:Tumor Necrosis Factor-Alpha.).

During myocardial infarction, specific subpopulations of CCR2^+^ CRMs are activated by damage-associated molecular patterns (DAMPs) through Toll-like receptors (TLRs) and their downstream myeloid differentiation primary response 88 (MyD88)-dependent signaling pathways, subsequently leading to the release of cytokines and chemokines (119–121). These results identify CCR2^+^CRMs as early central drivers of inflammation, whose activation directly triggers immune cell infiltration and myocardial injury. CRMs are generally recognized as crucial mediators of early adaptive responses in cardiomyocytes. The distinct roles of various CRMs in cardiac inflammation underscore the necessity of their harmonious coordination and equilibrium for the restoration of cardiac function (122, 123). Interactions among structural remodeling, inflammatory components, and electrical changes are pivotal in the pathophysiology of arrhythmias. The evolving understanding of tissue-resident macrophages uncovers their intricate, context-dependent roles in arrhythmia pathophysiology. Blocking the progression of fibrosis by completely inhibiting the occurrence of inflammation is detrimental to both cardiac and overall physiological health. A controlled degree of inflammation is essential for initiating tissue repair, necessitating a balanced regulation between pro-inflammatory and anti-inflammatory. Cumulative evidence indicates that augmenting CRM populations significantly attenuates pro-arrhythmic remodeling (**Figure 3**).

### 5.1 The dispute of existence and unsolved problems

In recent years, the role of CRMs in the pathogenesis of arrhythmias has gradually attracted the attention of researchers. However, despite considerable investigation, several mechanistic and conceptual issues remain unresolved or subject to debate.

First, the specific mechanism underlying the role of CRMs in cardiac electrophysiological changes remains incompletely understood. Some studies propose that CRMs may regulate the electrical activity of cardiomyocytes by secreting specific cytokines and signaling molecules. For example, CRMs modulate the function of calcium ion channels in cardiomyocytes by secreting pro-inflammatory factors such as IL-1β and TNF-α, thus altering the electrical activity of the heart. However, other studies have challenged this mechanism, proposing that the factors secreted by macrophages may exert their effects in a more indirect manner, requiring the involvement of additional intermediary pathways to influence the electrophysiological activity of the heart (124).

Second, the expression and activation of ion channels in CRMs warrant further investigation. It is worth noting that the contribution of calcium ion conduction channels to current activation is significantly less pronounced than that of potassium channels, while the function of chloride ion channels remains unclear. This observation provides a foundational basis and direction for further exploration of the existence and activation mechanisms of different selective ion currents (such as ligand-gated or mechanically-activated channels). Moreover, it contributes to addressing questions regarding the functions and electrophysiological regulation of both typical and atypical macrophages within healthy and diseased heart tissue (125, 126).

CRMs are also present in the cardiac conduction system, including the sinus node. How their presence affects the development and homeostasis of the cardiac conduction system, and whether it influences the development and function of the sinus node pacemaker remain worth further exploration. Age-dependent fibrotic replacement occurs in the SAN and AVN. Given that the distribution and function of CRMs may change with age, exploring whether age-dependent changes affect the common fibrosis in the SAN and AVN in the elderly represents a compelling starting point. In diabetic mice, CRMs secrete IL-1β to mediate the occurrence of ventricular arrhythmia. Experimental findings indicate that the stability of the electrical activity of cardiomyocytes is disrupted, although the specific mechanism remains unclear (127).

Using macrophage-reporting mouse lines combined with optical clearance technology and confocal microscopy, the author found that there are a large number of macrophages at the distal end of the AVN in mice and humans (84). Are there other undetermined paracrine mechanisms that allow CRMs to affect the heart rhythm in an atypical way? From a broader perspective, what role does atypical CRMs function play in the process of remodeling and neonatal regeneration after MI? Perhaps the most exciting aspect of the current research is the potential for targeted treatment of CRMs in various disease states (128). Using high-throughput scRNA sequencing, studies have identified the transcriptional and cellular diversity of normal human hearts. Identification of discrete cell subtypes and differentially expressed genes within the heart will ultimately facilitate the development of new therapies for cardiovascular diseases. In addition, cell therapy based on macrophages is also a direction worth exploring.

CRMs are directly coupled with cardiomyocytes, and their disturbances will change heart conduction. The specific mechanism remains to be explored. This suggests that the pharmacological modulation of CRMs may represent a new strategy against arrhythmia (129).

There may be other metabolites in the myocardial cell-macrophage interaction, which undoubtedly pave the way for the development of new therapeutic strategies that focus on regulating the function of macrophages in the arrhythmic heart. Studying the molecular and physiological regulation of CMs by macrophages is essential for understanding novel mechanisms behind heart disease from a systematic perspective and developing new treatment modalities (130).

### 5.2 What are the methodological limitations in current research on cardiac resident macrophages?

Current research on the role of CRMs in arrhythmogenesis faces critical methodological limitations. First, studies heavily rely on mouse models, but significant species differences exist between murine and human CRMs in terms of origin, subsets, and function. Moreover, obtaining high-quality, region-specific human heart samples is extremely challenging, severely hindering clinical translation (131, 132). Second, there is a notable “ventricle-centric bias” in the research focus. While extensive work concentrates on ventricular remodeling post-myocardial infarction, the role of CRMs in clinically more prevalent arrhythmias like atrial fibrillation remains understudied, leading to an incomplete understanding (132). On the technical front, surface markers (e.g., CCR2) used to distinguish CRM subsets dynamically change during pathology, affecting sorting purity and data interpretation (97). Furthermore, mainstream techniques, such as sequencing or patch-clamp electrophysiology performed on isolated cells, cannot achieve real-time, *in vivo* observation of the dynamic interactions between CRMs and other cardiac cells. Consequently, functional studies often remain at the correlative level (133). These limitations, stemming from model systems, research scope, and technical approaches, challenge the generalizability and clinical relevance of current findings. Future research must prioritize developing human-specific models and expanding investigations into atrial arrhythmias.

### 5.3 Future research direction and potential application prospects

The future research directions and potential application prospects regarding the electrophysiological mechanism and functional role of cardiac resident macrophages in arrhythmia mainly include the following aspects.

First, the molecular mechanisms of macrophages in the heart require deeper exploration. Although studies have revealed the role of macrophages in the electrophysiological system of the heart, their specific molecular mechanisms are still not completely clear. Future research should focus on analyzing the specific signaling pathways of these cells in myocardial electrical activity and how they affect the ion channels and gap junctions of myocardial cells. Through the use of scRNA sequencing technology and gene editing technology, specific genes and molecular pathways of macrophages in cardiac electrophysiological regulation can be more accurately revealed (122).

Second, the potential application of macrophages in the treatment of heart disease warrants exploration. Given that macrophages play an important role in heart repair and inflammatory response, they may become a new target for heart disease treatment. Future research should evaluate the feasibility of treating arrhythmias by regulating macrophage function. For example, strategies involving particular drugs or gene therapies to regulate the activity of macrophages could improve cardiac electrophysiological function and prevent arrhythmias (134).

Although macrophage-targeted therapies hold great potential, they may pose risks of immunosuppression, such as excessive inhibition of macrophage function leading to immune deficiency, disruption of immune homeostasis, or off-target effects. Therefore, it is essential to strike a balance between specific delivery efficiency and the maintenance of immune homeostasis. Efforts should focus on identifying more specific markers for macrophage subpopulations and combining immunomodulatory strategies to maximize therapeutic benefits while minimizing side effects. Additionally, through interdisciplinary exploration involving nanotechnology, immunology, and genetic engineering, macrophage-targeted therapies are expected to become a key pillar of next-generation immunotherapies.
